# Percutaneous Transplant Liver Biopsies: Does Biopsy Needle Gauge Matter?

**DOI:** 10.7759/cureus.94978

**Published:** 2025-10-20

**Authors:** Kiyon Naser-Tavakolian, Connie Liou, Abin Sajan, Asad Baig, Hannah Bae, Lindsay Young, John Filtes, Sidney Brejt, Joshua L Weintraub, Stephen P Reis

**Affiliations:** 1 Vascular and Interventional Radiology, Columbia University, New York, USA; 2 Vascular and Interventional Radiology, Memorial Sloan Kettering Cancer Center, New York, USA

**Keywords:** biopsy complications, core biopsy, liver biopsy, liver transplant, pathologic adequacy

## Abstract

Introduction

This study evaluates coaxial core needle size (17/18G vs 19/20G) and sample adequacy in liver transplant biopsies.

Materials and methods

This was an Institutional Review Board (IRB)-approved retrospective study of 397 percutaneous transplant liver biopsies performed over four years with 17G/18G or 19G/20G coaxial and core needles. Medical records were reviewed for needle size, number of cores, tract embolization, complications, and sample adequacy by pathology. Statistical significance was determined using a combination of t-tests and chi-squared tests with a significance level less than 0.05.

Results

There were 257 18G core biopsies and 140 20G biopsies. Sample inadequacy was noted in 3/257 of the 18G biopsies and 19/140 of the 20G biopsies (p<0.0001). The mean number of cores taken was 3.17±0.72 and 3.06±0.81 for 18G and 20G biopsies, respectively (p=0.14). There was no significant difference between the two groups regarding the number of cores taken in the adequate or inadequate samples. Tract embolization was performed in 353/397 biopsies. One clinically significant bleeding complication was noted in a procedure done with a 20G needle with two core biopsies. No other major complications were noted.

Conclusion

Random transplant liver biopsies performed with coaxial 17G/18G core biopsy needles are more likely to provide adequate results than biopsies performed with 19G/20G needles. The risk of complications in transplant liver biopsies is low.

## Introduction

Transplanted livers are followed closely by hepatologists and transplant surgeons after initial organ transplantation. They are followed with serial liver function tests and other periodic tests to ensure that any graft dysfunction is identified early and can be addressed as soon as possible to preserve graft function and guide therapy. Despite advances in immune modulation, rejection is still the most common complication, occurring in 15%-25% of cases [1]. Liver biopsies can provide a diagnosis in most cases (~90%) when patients have elevated liver function tests [1-4]. Biopsies are most commonly performed percutaneously, through a transjugular venous approach, laparoscopically, and via open surgery. The decision depends on clinical factors such as coagulation labs, platelet count, and the presence of ascites. Percutaneous and transjugular are the most common types of liver biopsies performed, whereas laparoscopic and open surgical biopsies are now less commonly performed [5]. Percutaneous random transplant liver core needle biopsies are often performed by interventional radiology or occasionally gastroenterology/hepatology, with core samples sent in formalin for analysis by pathology. The type and size of the needle are often chosen at the operator's discretion or based on equipment availability. Biopsy needles that are commonly used for liver biopsies come in various designs, including suction needles, coaxial side cutting, and cutting needles, both of which are often spring-loaded. The needles come in different lengths, ranging from 10 to 20 cm, and gauges, ranging from 16G to 20G. The most commonly used sizes in interventional radiology include a 17G introducer and 18G coaxial spring-loaded two-stage biopsy needle or a 19G introducer and 20G coaxial spring-loaded two-stage biopsy needle [6,7].

Currently, there are studies that emphasize the importance of obtaining adequate samples for analysis and have evaluated 18G alone, 18G versus 16G and 20G needles in native liver biopsies, as well as studying the safety of 18G spring-loaded needles alone in native and transplant liver biopsies [8,9]. However, there are no comparisons between 18G and 20G core biopsy needles in the literature for transplanted liver evaluation. In this study, we reviewed percutaneous transplant liver biopsies over a four-year period to assess sample adequacy as determined by pathology and complication rate for 18G vs. 20G spring-loaded two-stage deployment biopsy needles. This article was previously presented as a meeting abstract at the 2025 Society of Interventional Radiology Annual Scientific Meeting on April 1, 2025.

## Materials and methods

This was an Institutional Review Board (IRB)-approved retrospective study of 397 random transplant liver biopsies performed between January 2020 and August 2024, utilizing 17G/18G or 19G/20G coaxial and core two-stage spring-loaded needles. This research was conducted in accordance with the ethical principles outlined in the Declaration of Helsinki, which provides guidance on ethical standards for research involving human participants. Participant confidentiality and anonymity were maintained throughout, and data was handled in compliance with applicable data protection regulations.

Inclusion criteria were patients with prior liver transplants, older than 18 years of age, who underwent a random percutaneous liver biopsy between January 2020 and August 2024. A total of 397 biopsies met the inclusion criteria. Patient medical records were reviewed for needle size, number of cores/passes performed, tract embolization agent used, complications including bleeding requiring imaging or angiography and infection (abscess seen on post-procedure follow-up), and sample adequacy deemed by pathology in the final pathology report. Pathologist experience varied from three to 20 years with most samples being evaluated by gastrointestinal (GI)/liver-specific pathologists with 20 years of experience. On-site cytology was not used to determine sample adequacy. The number of portal tracts was seldom reported by our pathologists and thus could not be consistently recorded to compare. If the samples were inadequate, the number of portal tracts noted was documented (if present in the pathologist's report). Statistical significance was determined using a combination of t-tests and chi-squared tests with a significance level of less than 0.05.

Interventional radiologists, with experience ranging from two to 30 years, performed percutaneous random liver biopsies. Before the procedure, a history, physical exam, and routine laboratory studies were obtained and reviewed. A complete blood count and prothrombin time/international normalized ratio (INR) were obtained pre-procedure. If the patient had any imaging, this was also reviewed to determine whether the transplant was a whole liver, split liver, or left lateral segment for pre-procedural planning. The Society of Interventional Radiology's (SIR) guidelines were thresholds for pre-procedural laboratory values [10]. Platelet count >50,000/mL and an INR <1.8 were used as thresholds for the biopsies. Antiplatelet agents and anticoagulants were held for hours to days according to SIR guidelines depending on the patient's agent. If moderate sedation was planned for the procedure, the patients were advised to fast for a minimum of eight hours before the procedure. The procedures were performed at the main hospital or the freestanding office-based laboratory affiliated with the hospital.

The patient was positioned supine on the examination table or toward the right side of the bed if performed on the patient's stretcher. The patient's right arm was either placed above the head or on the chest. All procedures were performed under real-time ultrasound guidance. No real-time cytology or frozen pathologic evaluation was performed in these biopsies. Before sterile preparation, an ultrasound of the liver was performed to determine an ideal access site, with the decision to target the left or right lobe being determined by the operator and based on ultrasound visibility of the target. Once identified, the skin was prepped and draped in the usual sterile fashion. Local lidocaine (1 or 2%) was administered under live ultrasound guidance. Then, a small skin incision was made to allow for passage of the introducer needle (17G or 19G). The brand of the two-stage spring-loaded side-cutting core biopsy needle was either Temno (Merit Medical, South Jordan, UT, USA) or Bard Mission (BD, Franklin Lakes, NJ, USA), and it was selected based on operator preference. Needle throw distance was set to 2 cm for all samples obtained. Under ultrasound guidance, the needle was passed through the soft tissues and into the liver parenchyma. The introducer needle was positioned a few centimeters beyond the capsule of the liver within the parenchyma. Biopsies were performed using the coaxial two-stage spring-loaded needle (18G or 20G). A minimum of two samples were obtained under ultrasound guidance and placed directly into buffered formalin. After the samples were obtained, a tract embolization was performed depending on operator preference. Operators most commonly use gel foam slurry (gel foam mixed with sterile saline using a three-way stopcock). Other agents include gel foam pledgets (rolled small pieces of gel foam pushed out of the introducer needle using the inner stylet), autologous blood clot, thrombin, or no tract embolization agent. A small amount of the agent was given in the parenchyma in the needle's path, and the additional agent was often given at the capsule of the liver to promote hemostasis. After removing the needle, pressure was held at the access site for one to two minutes, and a sterile dressing was applied. If the right lobe of the liver was sampled, the patient was usually instructed to lie on the right side (right lateral decubitus) for one-hour post-procedure and then supine for another hour after the procedure. If the left lobe of the liver was sampled, then the patient was instructed to remain supine for two hours post-procedure. Vital signs were obtained per protocol every 15 minutes for the first hour and every 30 minutes for the second hour. The patients were typically monitored for anywhere from two to four hours post-procedure and discharged from the department if they felt well and had no concerning signs of complications. Postoperatively, the patients were instructed to avoid heavy lifting (>10 lb) for three days. If the patient had worsening pain in the right upper quadrant, hypotension, or tachycardia, the operator was notified, and further imaging (CT angiography) or conservative management was performed at their discretion.

Statistical analysis

Data was analyzed using IBM SPSS Statistics, version 26 (IBM Corp, Armonk, NY). Continuous variables were expressed as mean±standard deviation (SD) with 95% confidence intervals, and categorical variables were presented as counts (N) and percentages (%). Comparisons between groups for continuous variables were performed using independent t-tests for normally distributed data. Categorical variables were compared using chi-squared tests. A p-value of less than 0.05 was considered statistically significant. Test statistics (t-value for t-tests, χ^2^ for chi-squared tests) are reported alongside p-values to facilitate interpretation.

## Results

There were 233 male and 164 female patients with a mean age of 52 years (±15.6). No significant difference between male and female ages was noted (t=0.87, p=0.39). There were 257 biopsies performed using an 18G core biopsy needle and 140 biopsies performed using a 20G biopsy needle for a total of 397 biopsies. The mean number of cores taken was 3.17 (±0.72, 95% CI: 3.08-3.26) and 3.06 (±0.81, 95% CI: 2.92-3.19) for 18G and 20G biopsies, respectively (t=1.48, p=0.14). No repeat biopsies were performed for inadequate samples within a two-week period. 

Samples were considered adequate by pathology in 254 of 257 (98.8%) biopsies performed using an 18G needle and in 121 of 140 (86.4%) biopsies using a 20G needle (χ^2^=25.67, p<0.0001). Additionally, there was no difference in the number of cores taken for adequate or inadequate samples between the 18G and 20G groups (t=1.15, p=0.25 for adequate; t=0.52, p=0.60 for inadequate). The right lobe of the liver was biopsied in 333 of 397 (83.8%) cases (230 with 18G, 103 with 20G), and the left lobe was biopsied in 64 of 397 (16.1%) cases (28 with 18G, 36 with 20G). Pathologic adequacy of the 18G needles was noted in 227/230 (98.6%) biopsies of the right hepatic lobe (χ^2^=21.34, p<0.0001) and 28/28 (100%) biopsies of left hepatic lobe (χ^2^=5.11, p=0.02). Comparatively, adequacy for 20G needles was 90/103 (87.4%) for right lobe and 30/36 (83.3%) for left lobe. For inadequate cases, portal tracts were reported in two out of three 18G biopsies (mean 3.5±2.1) and 14 out of 19 20G biopsies (mean 4.0±1.5) (t=0.25, p=0.80). Additional data from 50 randomly selected adequate samples (25 per group) showed a mean of 12.3±2.1 portal tracts for 18G and 10.8±2.5 for 20G (t=2.35, p=0.02). These findings are summarized in Table 1.

**Table 1:** Comparison of 18G vs. 20G Transplant Liver Biopsies. Data are presented as N (%), mean±SD with 95% confidence intervals where applicable. Statistical significance was determined using t-tests for continuous variables and chi-squared tests for categorical variables, with p<0.05 considered significant. G: gauge (needle).

	18G	20G	χ^2^ value	t value	p-value
Number of biopsies (N, %)	257 (64.7%)	140 (35.3%)			
Mean number of cores taken (mean±SD, 95% CI)	3.17±0.72 (3.08-3.26)	3.06±0.81 (2.92-3.19)		1.48	0.14
Right-lobe biopsies (N, %)	230 (69.1%)	103 (30.9%)			
Left-lobe biopsies (N, %)	27 (42.8%)	36 (56.2%)			
Pathology adequacy rates (N, %)	254/257 (98.8%)	121/140 (86.4%)	25.67		<0.0001
Right-lobe adequate rate (N, %)	227/230 (98.6%)	90/103 (87.4%)	21.34		<0.0001
Left-lobe adequate rate (N, %)	28/28 (100%)	30/36 (83.3%)	5.11		0.02
Pathology adequate (mean cores±SD)	3.17±0.71	3.08±0.79		1.15	0.25
Pathology inadequate (mean cores±SD)	3.33±1.52	3.00±0.94		0.52	0.60
Mean number of portal tracts in inadequate samples (mean±SD)	3.5±2.1 (N=2/3)	4±1.5 (N=14/19)		0.25	0.80

Tract embolization was performed in 353 of 397 (88.9%) biopsies. Gel foam slurry was used in 330 (83.1%) cases, an autologous blood clot was used in 20 (5.0%) cases, gel foam pledgets were used in two (.5%) cases, and thrombin was used in one case. Only one case of sizeable subcapsular/intraparenchymal hematoma with hemodynamic instability was noted. This patient had a right-lobe biopsy with a 20G needle with two cores obtained and a gel foam slurry used for tract embolization. Due to their hemodynamic instability and imaging showing subcapsular hematoma, they underwent angiography, demonstrating a 1.5 cm pseudoaneurysm within segment VIII. This patient underwent embolization of the pseudoaneurysm using gel foam and coils and had a short hospital stay with subsequent discharge. No other major complications were noted.

## Discussion

Pathologic adequacy is crucial for accurate and timely histologic assessment during liver biopsy after a liver transplant. Pathologists use specific standardized scoring systems and guidelines to assess the degree of liver injury and rejection. Features such as portal inflammation, bile duct damage, and endothelial inflammation are part of the Banff rejection activity index and are used to identify signs of rejection. They require a minimum number of portal tracts on the biopsy specimen to accurately assess these three categories and provide a clear assessment of the transplanted liver [11,12]. This varies significantly from biopsies performed for liver masses, evaluating for primary, benign, or metastatic disease.

In the literature, no studies to date compare the use of 17G/18G and 19G/20G coaxial systems for transplant liver biopsies. Prior studies have stated the safety and efficacy of using a 17G/18G system or 18G only biopsy system in native and transplant livers. A study by Eraslan et al. evaluated 18G vs 20G biopsies in liver masses specifically and found no difference in sample adequacy rates between the two biopsy needles [13]. However, they had a smaller sample size (60 biopsies), and the study focused on masses instead of normal liver parenchyma. Additionally, a study performed in 2001 by Röcken et al. demonstrated no significant difference in adequacy rates when comparing 17G, 20G, and 21G needle biopsies. However, they found a significant difference in the number of portal tracts between the needle sizes, with the 17G providing the greatest number of portal tracts and 21G providing the least number of portal tracts [14]. Again, this study differs from ours as it studied native liver biopsies instead of transplanted liver biopsies. A randomized controlled trial study by Jing et al. analyzed 300 biopsies in 282 pediatric patients with transplanted livers who were assigned to undergo a 16G or 18G needle biopsy. They found that the 16G needle group had more complete portal tracts than the 18G group (20 vs 18 portal tracts, respectively) but no significant difference in specimen inadequacy rate [15]. A similar randomized controlled study by Tublin et al. in the *American Journal of Roentgenology* compared 16G and 18G biopsies in native livers and found a significant difference in 16G vs 18G biopsies concerning portal tracts (14 vs 13 tracts, respectively) and found lower adequacy rates with both needles compared to our study, 85% for 16G and 80% for 18G, respectively, with no significant difference between the two groups [16]. Our study found a statistically significantly increased number of inadequate biopsy samples when using a 19G/20G coaxial system versus a 17G/18G coaxial system, with no significant difference in the average number of cores taken in either group. Although an adequacy rate of 86.4% in the 19G/20G group is high, we did not note increased complications when using 17G/18G systems and reported a much higher 98.8% adequacy rate. In contrast, studies on native liver biopsies such as Shah et al. demonstrated that 19G needles significantly outperform 22G needles in endoscopic ultrasound (EUS)-guided biopsies, which despite being a different technique, found that the 19G needles provided longer cores and more portal tracts as one would expect. This likely reflects the greater need for comprehensive histological assessment in native livers (e.g. fibrosis staging), whereas transplant livers often have prioritized specific findings such as rejection, which may need fewer tracts as supported by Jing et al. [15,17]. There is scarcity of data on direct native vs. transplant liver biopsy comparisons and this would be an interesting point for future research, Also, of interest, there was approximately a 5% difference between pathologic adequacy rates when comparing all (18G and 20G) right lobe and left lobe biopsies, but this failed to reach statistical significance, likely due to only 22 of the 397 samples being considered inadequate.

The only major complication in the study occurred within the 19G/20G group. This was hemodynamically significant bleeding, which required embolization. Interestingly, only two cores were taken in that instance, and gel foam slurry embolization of the tract was performed before needle withdrawal. This resulted in a major complication rate of 0.25%. Most of our operators use some form of tract embolization, and no complications from tract embolization were noted post-procedure. As such, we do not see a disadvantage to some form of tract embolization in transplant liver biopsies; however, further direct comparison studies can better elucidate the potential benefits and differences.

The limitations of our study include the retrospective nature of this study performed at a single center, variable operators performing the biopsies with varying levels of experience, lack of number of portal tracts being routinely reported on our pathology reports, and non-randomization with regard to tract embolization. Additionally, on-site cytology was not used in this study which can alter adequacy rates as additional cores can be taken if cytology deems a specimen inadequate on touch prep. Also, it is unclear why some operators preferred a 17G/18G coaxial system versus a 19G/20G coaxial system which may contribute to selection bias.

## Conclusions

The findings of our study are important for the field of transplant liver biopsies. We have demonstrated that random transplant liver biopsies performed with 17G/18G spring-loaded two-stage deployment coaxial and core biopsy needles with three core samples are more likely to provide adequate samples than three core samples from 19G/20G biopsy needles. This study supports using 17G/18G coaxial systems with three cores taken for transplant biopsies to provide the highest chance for adequate specimens. Further multi-center studies can provide additional validation to these findings. The risk of major complications in transplant liver biopsies is low. Tract embolization may lower the risk of complications, but further comparison studies with larger sample sizes are needed to fully understand its benefits.
